# Mucocele in Lower Lip as a Result of Improper Use of Feeding Bottle: A Case Report

**DOI:** 10.1155/2013/520425

**Published:** 2013-03-19

**Authors:** S. Ashok Kumar, Mahesh Ramakrishnan

**Affiliations:** ^1^Department of Periodontics, Madha Dental College, Somangalam Road, Kundrathur, Chennai 600 069, India; ^2^Department of Pediatric Dentistry, Madha Dental College, Somangalam Road, Kundrathur, Chennai 600 069, India

## Abstract

A rare case of mucocele associated with improper feeding habit has been presented. An eight-month old male child presented with swelling in lower lip which was noticed by his mother a week earlier. A thorough clinical examination and history taking gave a diagnosis of mucocele resulting from improper use of feeding bottle. This case highlights and discusses the history, the clinical along with histologic features, and the clinical management of this lesion. Awareness of such an entity and the functional problems associated with the lesion will help the pediatric dentist to prevent any further complications.

## 1. Introduction 

Mucocele is the common minor salivary gland lesion, which is clinically characterized by a single or multiple, spherical, and fluctuant nodules which are generally asymptomatic [1]. They are the 15th most common oral mucosal lesion, with a prevalence of approximately 2.4 cases per 1,000 people [2]. A study by Jones and Franklin, considering 4,406 pediatric patients, showed that the most common lesion was mucocele (16%) [3]. The clinical presentation depends upon the depth within the soft tissue and the degree of keratinization of the overlying mucosa. Superficial lesions present as raised soft tissue swelling having bluish color, while the deeper lesions are more nodular, lack the vesicular appearance, and have a normal mucosal color. In certain cases the diagnosis may require advanced diagnostic methods to better visualize form, diameter, and position of the lesion relative to adjacent organs [4]. 

Yamasoba et al. [5] highlight two crucial etiological factors in mucocele: traumatism and obstruction of salivary gland ducts. While the most common age group is in the second decade of life, there are also case reports of mucocele present in neonate [6, 7]. Although most of the cases are asymptomatic, functional disturbances such as difficulty in feeding and dietary habits may require immediate surgical intervention. 

The present case reports the clinical and histopathologic findings of a lower lip mucocele in an 8-month-old child associated with an improper use of feeding bottle.

## 2. Case Report 

**Figure 1:**
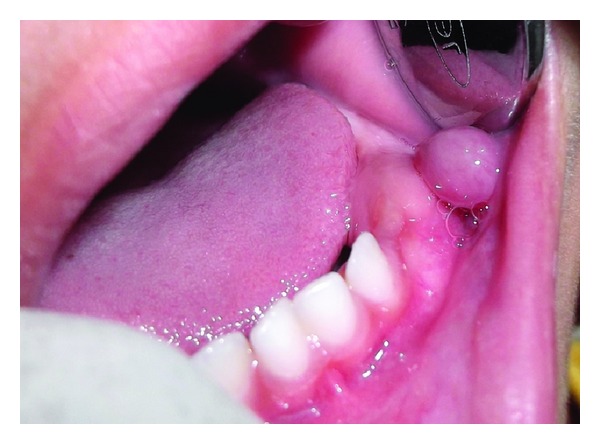
Clinical presentation of the lesion.

An eight-month-old male child was brought to our Department by his mother with the chief complaint of a swelling on the lower lip for the past one week. The mother gave a history that the child has been weaned from breast feeding two months before and currently using the feeding bottle, which the child used to bite hard and kept in mouth most of the day time. The parent reported difficulty in feeding due to the presence of swelling. Intraoral examination revealed presence of upper and lower primary incisors, and a localized, compressible, and soft fluid-filled nodule of approximately 8 × 8 mm diameter 0 (Figure 1) with a translucent to blue surface seen in the lower buccal mucosa corresponding to the canine region. 

**Figure 2:**
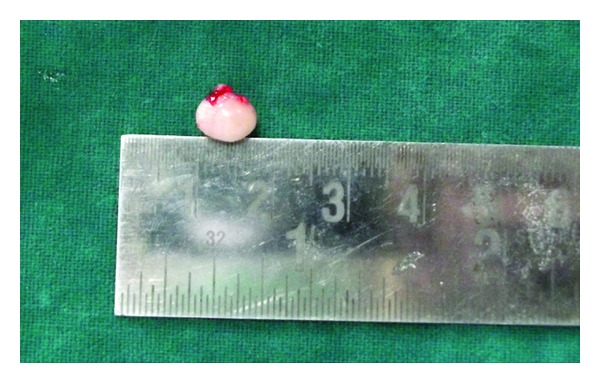
The excised mucocele tissue.

**Figure 3:**
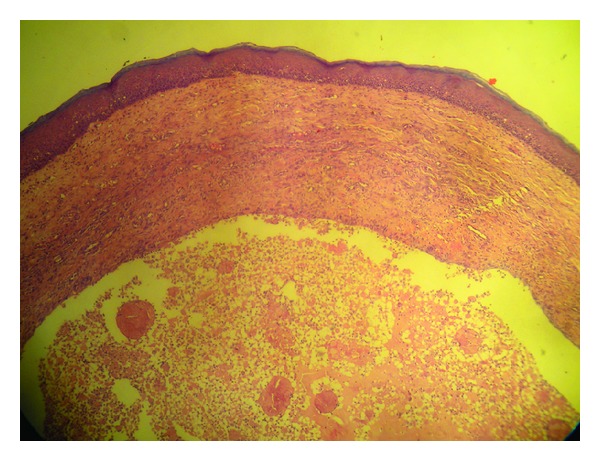
Histopathological analysis of the excised tissue showing the stratified squamous epithelium with underlying mucoid material and inflammatory cells.

**Figure 4:**
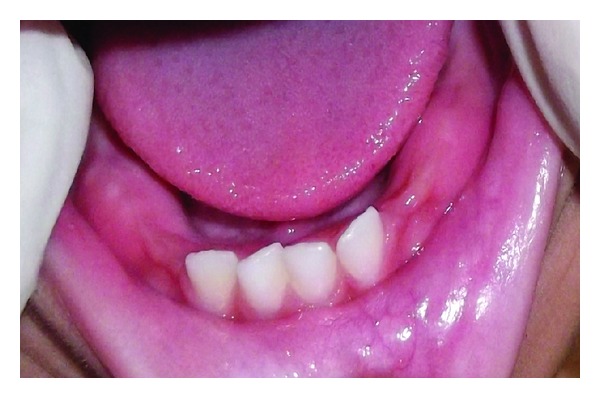
1 week postoperatively showing normal healing.

Considering the size of the swelling and the functional disturbances associated with it, the treatment plan was aimed for a complete surgical excision of the lesion. Excision of the mucocele was performed under local anesthesia, and the wound was sutured using a resorbable suture polyglactin 910 (Vicryl). The excised tissue was subjected to histopathological examination, which showed a mucous excavation cyst, lined by an epithelium (Figures 2 and 3). The child reported uneventful recovery and an improved dietary habit one week postoperatively (Figure 4).

## 3. Discussion 

A mucocele or mucous cyst is a common, mucus-containing cystic lesion of the minor salivary glands in the oral cavity. Some authors prefer the term mucocele since most of these lesions are not true cysts due to the absence of epithelial lining [2]. 

The literature review had cited the reason for some of the oral lesions like irritation fibroma and mucocele arising as a result of oral habits such as lip biting/sucking [8]. During the tooth eruption period, the oral tissues become very sensitive and children try to relieve the eruptive symptoms by biting the pacifier/feeding bottle with exaggerated force and sometimes in the wrong place, leading to the development of a wide spectrum of pathologies. In the present case the child must have been using the bottle to be relieved from the discomforts associated with teething, and also the early eruption of the dentition may have been a contributing factor for the suckling trauma. Children often show signs of teething characterized by increased salivation, fever, and chronic irritation which should be considered, and adequate care during this period can prevent complications. This indicates the need for creating awareness among the parents about the teething process and the proper management. 

Mínguez-Martinez et al. [9] reviewed 89 patients with the mean age of 6.1 years. They noted that the average progression time for the mucocele includes 5.7 months. It was observed that older patients developed more mucoceles on the tongue and lips and younger patients developed more mucoceles on the buccal mucosa and palate. 

The present case is an extravasation type of lesion, which has a tendency to occur in younger patients, whereas the second type retention mucoceles may occur most often in middle to late life. Few cases have been reported in the first decade of life and their occurrence in new-born babies has rarely been reported [6, 7]. 

The main area of controversy surrounding the surgical excision of mucocele in very young children is related to the anesthetic modality and the degree of cooperation during the surgical excision. In most of the reported cases the lesion was excised under general anesthesia [6, 7]. The option for general anesthesia is usually related to the patients' age and is irrespective of the size of the lesion. While most of the cases are managed under surgical excision, there are case reports of the use of laser ablation, cryosurgery, and electrocautery approaches that have also been used for the treatment of the conventional mucocele with variable success [1]. 

The present case illustrates the importance of having a complete knowledge of the lesion and the need for careful history, which will in many cases lead to the diagnosis of main etiology for the lesion. Surgical removal was necessary in this case because of the associated functional problems, which is confirmed by the improvement in dietary habits postoperatively.
